# Best Practices for Handling and Administration of Expressed Human Milk and Donor Human Milk for Hospitalized Preterm Infants

**DOI:** 10.3389/fnut.2018.00076

**Published:** 2018-09-03

**Authors:** Caroline Steele

**Affiliations:** Children's Hospital of Orange County, Orange, CA, United States

**Keywords:** human milk handling, infant feeding preparation, human milk bar code scanning, aseptic technique feeding preparation, safety and human milk

## Abstract

The importance of human milk for the preterm infant is well established (1–3). However, the feeding of human milk to preterm infants is typically much more complicated than the mere act of breastfeeding (3, 4). The limited oral feeding skills of many preterm infants often results in human milk being administered via an enteral feeding tube (4). In addition, fortification is commonly required to promote optimal growth and development—particularly in the smallest of preterm infants (2, 4, 5). Consequently, a mother's own milk must be pumped, labeled, transported to the hospital, stored, tracked for appropriate expiration dates and times, thawed (if previously frozen), fortified, and administered to the infant with care taken at each step of the process to avoid microbial contamination, misadministration (the wrong milk for the wrong patient), fortification errors, and waste (1–5). Furthermore, the use of pasteurized donor human milk (DHM) for preterm infants when a mother's own milk is not available has been endorsed by many organizations (1). Therefore, appropriate procurement, storage, thawing (if received frozen), fortification, labeling, and administration must occur with the same considerations of preventing contamination and fortification errors while ensuring the correctly prepared final product reaches the correct patient (1). Many professional organizations have published best practices to provide hospitals with guidelines for the safe and accurate handling and preparation of expressed human milk (EHM) and DHM feedings for preterm infants (1–5). These best practices emphasize the importance of preparation location, trained staff, proper identification of human milk to prevent misadministration, and strategies to prevent fortification errors (1–6). The purpose of this mini-review article is to summarize current published best practices for the handling of human milk for preterm infants within the hospital setting (1–6). Emphasis will focus on the use of aseptic technique with proper sanitation and holding times/temperatures to limit microbial growth; use of technology to prevent misadministration of human milk and fortification errors as well as for tracking of expiration dates/times and lot numbers; and workflow strategies to promote safety while improving efficiencies (1–7).

## Introduction

The importance of human milk for the preterm infant is well established (1–3). However, the feeding of human milk to preterm infants is typically much more complicated than the mere act of breastfeeding (3, 4). The limited oral feeding skills of many preterm infants often results in human milk being administered via an enteral feeding tube (4). In addition, fortification is commonly required to promote optimal growth and development—particularly in the smallest of preterm infants (2, 4, 5). Consequently, a mother's own milk must be pumped, labeled, transported to the hospital, stored, tracked for appropriate expiration dates and times, thawed (if previously frozen), fortified, and administered to the infant with care taken at each step of the process to avoid microbial contamination, misadministration (the wrong milk for the wrong patient), fortification errors, and waste (1–5).

Furthermore, the use of pasteurized donor human milk (DHM) for preterm infants when a mother's own milk is not available has been endorsed by many organizations including the World Health Organization (WHO), the Academy of Breastfeeding Medicine (ABM), the European Milk Bank Association, the Human Milk Banking Association of North America (HMBANA), the European Society for Pediatric Gastroenterology, Hepatology, and Nutrition (ESPGHAN), the American Society for Parenteral and Enteral Nutrition (ASPEN), the United States Surgeon General, the Academy of Nutrition and Dietetics, and the American Academy of Pediatrics (AAP) (1). Therefore, appropriate procurement, storage, thawing (if received frozen), fortification, labeling, and administration must occur with the same considerations of preventing contamination and fortification errors while ensuring the correctly prepared final product reaches the correct patient (1).

Many professional organizations, including the Academy of Nutrition and Dietetics, ASPEN, the National Association of Neonatal Nurses (NANN), and HMBANA, have published best practices to provide hospitals with guidelines for the safe and accurate handling and preparation of expressed human milk (EHM) and DHM feedings for preterm infants (1–5). These best practices emphasize the importance of preparation location, trained staff, proper identification of human milk to prevent misadministration, and strategies to prevent fortification errors (1–6).

The purpose of this mini-review article is to summarize current published best practices for the handling of human milk for preterm infants within the hospital setting (1–6). Emphasis will focus on proper sanitation, use of technology for tracking and error prevention, and workflow strategies to promote safety while improving efficiencies (1–7).

## Location

For handling of human milk, fortifiers, and feeding systems, preparation location and practices that minimize microbial growth (such as adherence to good hand-hygiene practices and use of “no touch” preparation and administration techniques) are critical (1). A location dedicated for the purpose of handling human milk feedings that is separate from patient care areas reduces risk of contamination and is considered a best practice (1, 2, 8, 9). EHM or DHM feedings should not be prepared in *any* patient care area, including the patient's bedside, due to risk of contamination (1, 2, 5, 7, 9).

## Equipment and supplies

### Sinks and dishwashers

The preparation area should contain a handwashing sink with hands-free controls (1). Unless all preparation items are disposable, a three-compartment sink or commercial dishwasher is needed to ensure proper cleaning and sanitizing of all reusable items (1, 10, 11). The dishwasher should reach a wash temperature of 66°C (150°F) and a rinse temperature of 82°C (180°F) (10, 11).

### Refrigerators and freezers

Although not required, dedicated human milk refrigerators and freezers are preferred. Adequate space to store human milk while allowing for appropriate airflow is important to ensure proper temperatures. Refrigeration guidelines for the storage of human milk for healthy infants at home have been described (12). Within the health care setting, refrigerators for human milk storage must be able to maintain temperatures between 2–4°C (35–39°F); freezers must allow for temperatures at or below −20°C (−4°F) to long-term storage (1, 13). A reliable method of temperature monitoring is imperative to prevent loss and promote safety (1). Use of automated systems that alarm when temperatures exceed desired ranges may be beneficial. Location of refrigeration units in areas with limited access, may help prevent tampering and waste.

### Laminar flow hoods

While laminar flow hoods provide an additional barrier against contaminants, they are typically used in the preparation of sterile products (including medications and processing/packaging of pasteurized donor human milk) (1, 14). However, use of a flow hood does not result in a sterile finished product when used during the preparation of non-sterile feedings (such as unpasteurized EHM and/or non-sterile fortifiers or additives) (1, 15). Furthermore, use of a flow hood should not be a replacement for good handing practices and aseptic technique.

### Measuring/mixing devices and storage containers

All preparation and storage items should be made of stainless steel or food grade plastic that is free of bisphenol A (BPA) and Di(2-ethylhexyl) phthalate (DEHP) (11). Glass items (such as graduated cylinders or beakers) are not generally used for routine handling of human milk in the health care setting due to risk of exposure to glass particles should the glass crack or break (11).

Single-use, disposable items are often selected for human milk collection and feeding preparation due to their convenience and sanitation. Such items may be sterile or non-sterile as there is no evidence that use of non-sterile items results in higher bacterial loads in collected human milk or prepared feedings (1, 16). If reusable items are selected, they must be cleaned and appropriately sanitized between uses to prevent cross contamination.

Human milk and other liquid ingredients should be measured using containers with precise graduations such as graduated cylinders, beakers, liquid measuring cups, or syringes (1). Powdered fortifiers and additives should be measured on a gram scale accurate to a tenth of a gram (1). Scales should undergo regular calibration to ensure accuracy and promote safety (1).

## Staffing and staff hygiene

Use of dedicated staff for the handling and preparation of human milk feedings within the health care setting is considered a best practice and has been shown to reduce risk of misadministration errors (1–3, 5). Staff should be well trained in aseptic technique and demonstrate proper steps for handling human milk and fortifiers. Hand hygiene is critical in the handling of human milk to prevent introduction of exogenous microbial contamination (17, 18).

Use of disposable gowns and other personal protective items including a bonnet or hairnet and gloves are recommended (1). Artificial nails and long natural nails have been associated with a *Pseudomonas aeruginosa* outbreak in a neonatal intensive care unit (18, 19). Therefore, it is recommended that staff nails should be short, neatly groomed, and unpolished (17–21).

## Human milk storage

Stored milk should be rotated using first-in-first-out (FIFO) principles with the oldest milk being used first. Storage times and temperatures impact nutritional quality, biologically active components in human milk, and rate/incidence of microbial growth (12, 22–26). Within the acute care setting when human milk is used for immunocompromised patients, storage recommendations are more conservative than for the healthy infant at home (1, 12). Therefore, it is generally recommended (1, 13, 16, 22, 27):

- Fresh milk be stored in the refrigerator (≤ 4°C or ≤ 39°F) for a maximum of 48 h
- Thawed unpasteurized milk be stored in the refrigerator (≤ 4°C or ≤ 39°F) for a maximum of 24 h
- Thawed pasteurized DHM be stored in the refrigerator (≤ 4°C or ≤ 39°F) for a maximum of 48 h
- Fortified milk be stored in the refrigerator (≤ 4°C or ≤ 39°F) for a maximum of 24 h
- Hang time for continuous feedings at room temperature for a maximum of 4 h
- Frozen human milk be stored in the freezer for 6–12 months at ≤ −20°C (≤ −4°F) or beyond 12 months at −70 to −80°C (−94 to −112°F).

## Preparation and administration of human milk feedings in the health care setting

Handling of human milk and preparation of individual feedings within the health care setting requires strict adherence to guidelines to ensure the preservation of nutrients and bioactive compounds while reducing risk of harmful microbial growth (1). Fortification accuracy is imperative to prevent feeding intolerance and promote optimal health and growth. Steps for human milk feeding preparation within the acute care setting are outlined in Table 1 (1, 13, 28–32).

**Table 1:** Steps for human milk feeding preparation within the acute care setting (1, 13, 28–32).

Don personal protective items per facility policy (may include disposable gowns and bonnets/hairnets)
Perform hand hygiene upon entry into the preparation area, after sanitizing work surfaces, and between each individual patient feeding preparation
Sanitize work space using a facility-approved sanitizing solution appropriate for food contact surfaces upon entry, between each individual patient feeding preparation, and as required to support aseptic technique
Thaw milk if needed using water bath or commercial warmer
Perform a two-person double check of a minimum of two-patient identifiers or use bar code scanning technology to confirm that all bottles of human milk belong to the same patient before combining
Following hand hygiene, don gloves prior to initiating the actual preparation
Measure appropriate volume of human milk using measuring container with 1 mL graduations
Add fortifiers, if appropriate
•Ensure accuracy with calculations and measurements to avoid over or under fortification
•Consider systems such as a two-person double check or bar code scanning to confirm appropriate fortifier is used
•Use pre-portioned fortifiers when available
•If not pre-portioned, measure liquid fortifiers using graduated cylinders, beakers, liquid measuring cups, or syringes and weigh powders using a gram scale
Gently mix ingredients in clean disposable or cleaned and sanitized reusable container
Place finished product in a clean disposable or cleaned and sanitized reusable closed container
•Prepare no more than 24-h volumes
•Finished product may be unit dosed for individual feedings or in bulk volumes
Label each container Recommended components include:
•Patient name
•Identification number (such as medical record number)
•Contents (human milk plus any fortifiers or additives)
•Caloric density
•Volume in container
•Volume per feeding and frequency or rate of administration
•Administration route
•Expiration date and time
•“For enteral use only” or “Not for intravenous use”
•“Refrigerate until use”
Refrigerate final product until used
Perform a two-person double check of a minimum of two-patient identifiers or use bar code scanning technology to verify the feeding label against the patient armband to confirm correct identity prior to administration
Monitor time for prepared feedings at room temperature
•Decant no more than 4-h volumes for continuous enteral feedings
•For oral feeding, discard any milk remaining in the bottle 1 h after initiating feeding due to potential for bacterial contamination from oral flora that may colonize the milk remaining in the bottle

Sterile liquid fortifiers and additives are preferred over powdered products (which are not sterile) to reduce the risk of microbial contamination; sterile options should be used for human milk fortification whenever possible (1, 13). At present, the optimal length of time between preparation and feeding of fortified human milk is unknown (13). Research has shown that over time, the osmolality of fortified human milk increases (by up to 4%) and the size of milk fat globules may become altered (possibly impacting fat digestion) (33). While shortening the storage time for fortified human milk may be advantageous, there is not enough published evidence to suggest a revision of the current recommendations for a maximum of 24 h (1, 13). Centralized fortification of human milk is a best practice and has been shown to improve patient safety (1–8, 13). However, centralized handling processes often preclude the ability to prepare each individual feeding immediately prior to use. Based on current evidence, the benefits of centralized handling appear to outweigh the risks of potential changes to human milk when feedings are prepared in advance (1–8, 13, 34, 35). Facilities may want to consider the shortest amount of time realistically feasible while still utilizing centralized handling processes. To this end, some organizations have opted to prepare 12-h volumes instead of 24-h volumes which also may be beneficial in more quickly implementing feeding order changes and preventing waste (1).

In addition to safe handling practices, processes must be in place to ensure safe administration of human milk and prevent inadvertent infusion via intravenous (IV) lines (1, 13). Enteral feeding misconnections, which may result in death, have been reported in the literature (36). The International Standards Organization (ISO) has set a standard for enteral devices to provide a female (administration set or syringe) to male (feeding tube) orientation known as ISO 80369-3 (37). Feeding connection sets with this unique configuration are known as ENFit® systems (1, 37). Adoption of ENFit® compatible connectors for all enteral infusions promotes patient safety by preventing enteral feedings from being accidentally connected to IV lines or other medical device ports (1, 13, 37).

## Use of bar code scanning technology to improve safety

Bar code scanning technology is commonly used in the health care setting to promote patient safety by reducing the risk of misadministration (providing the wrong product to the wrong patient) for processes such as medication, blood, and human milk administration (3–5). Bar code scanning is often used in lieu of a two-person double check to reduce risk of human error and confirmation bias which may occur when a manual check is used (3–5). Such systems have been shown to reduce errors and improve efficiencies (3–5). Scanning technology can assist with monitoring expiration dates and times. Human milk that is beyond its expiration is at greater risk for excessive microbial growth which could be particularly devastating in the critically ill neonate. Consequently, scanning systems may add a layer of patient safety by alerting the clinician if an attempt is made to use an expired feeding. Furthermore, some systems offer the ability to automate fortification calculations and scan fortifiers or additives to reduce risk of fortification errors (3–5). Automatically tracking lot numbers for pasteurized DHM and fortifiers or additives is more efficient than having staff track such information manually and provides a faster method of identifying exactly which patients received a particular product in the event of a product recall. Therefore, bar code scanning technology with human milk preparation and feeding is considered a best practice and is endorsed by many organizations (1–5).

## Summary

Human milk in the health care setting, particularly the neonatal intensive care unit, is often viewed as “medicine” or an adjunct therapy. Some of the most fragile patients are those premature or critically ill infants receiving human milk feedings. Therefore, every precaution must be taken with human milk handling to ensure safety. Aseptic technique with proper sanitation and holding times/temperatures to limit microbial growth; use of technology to prevent misadministration of human milk and fortification errors as well as for tracking of expiration dates/times and lot numbers; and workflow strategies to promote safety while improving efficiencies are worthy endeavors of all facilities (1–7).

## Author contributions

The author confirms being the sole contributor of this work and approved it for publication.

### Conflict of interest statement

The author declares that the research was conducted in the absence of any commercial or financial relationships that could be construed as a potential conflict of interest.
